# Modified-Live Feline Calicivirus Vaccination Reduces Viral RNA Loads, Duration of RNAemia, and the Severity of Clinical Signs after Heterologous Feline Calicivirus Challenge

**DOI:** 10.3390/v13081505

**Published:** 2021-07-30

**Authors:** Andrea M. Spiri, Barbara Riond, Martina Stirn, Marilisa Novacco, Marina L. Meli, Felicitas S. Boretti, Imogen Herbert, Margaret J. Hosie, Regina Hofmann-Lehmann

**Affiliations:** 1 Clinical Laboratory, Department of Clinical Diagnostics and Services and Center for Clinical Studies, Vetsuisse Faculty, University of Zurich, 8057 Zurich, Switzerland; briond@vetclinics.uzh.ch (B.R.); mstirn@vetclinics.uzh.ch (M.S.); mnovacco@vetclinics.uzh.ch (M.N.); mmeli@vetclinics.uzh.ch (M.L.M.); rhofmann@vetclinics.uzh.ch (R.H.-L.); 2 Clinic for Small Animal Internal Medicine, Vetsuisse Faculty, University of Zurich, 8057 Zurich, Switzerland; fboretti@vetclinics.uzh.ch; 3 Medical Research Council-University of Glasgow Centre for Virus Research, Glasgow G61 1QH, UK; imogen.herbert@glasgow.ac.uk (I.H.); margaret.hosie@glasgow.ac.uk (M.J.H.)

**Keywords:** feline calicivirus, clinical scoring, shedding, RT-qPCR, experimental infection, acute phase protein reaction, serum amyloid-A, haematology, lymphopenia, immune evasion

## Abstract

Feline calicivirus (FCV) is a common cat virus causing clinical signs such as oral ulcerations, fever, reduced general condition, pneumonia, limping and occasionally virulent-systemic disease. Efficacious FCV vaccines protect against severe disease but not against infection. FCV is a highly mutagenic RNA virus whose high genetic diversity poses a challenge in vaccine design. The use of only one modified-live FCV strain over several decades might have driven the viral evolution towards more vaccine-resistant variants. The present study investigated the clinical signs, duration, and amount of FCV shedding, RNAemia, haematological changes and acute phase protein reaction in SPF cats after subcutaneous modified-live single strain FCV vaccination or placebo injection and two subsequent oronasal heterologous FCV challenge infections with two different field strains. Neither clinical signs nor FCV shedding from the oropharynx and FCV RNAemia were detected after vaccination. After the first experimental infection, vaccinated cats had significantly lower clinical scores, less increased body temperature and lower acute phase protein levels than control cats. The viral RNA loads from the oropharynx and duration and amount of RNAemia were significantly lower in the vaccinated animals. No clinical signs were observed in any of the cats after the second experimental infection. In conclusion, FCV vaccination was beneficial for protecting cats from severe clinical signs, reducing viral loads and inflammation after FCV challenge.

## 1. Introduction

Feline calicivirus (FCV) is one of the most common viral pathogens in domestic cats worldwide [1]. Especially in multicat situations such as shelters or breeding catteries, FCV prevalence can range from low to high (10–90%) [2,3,4,5,6,7]. FCV is a single-stranded RNA virus with a positive polarity, belonging to the family Caliciviridae and the genus Vesivirus [1]. FCV is highly resistant to inactivation due to the lack of an envelope; therefore, infection of susceptible hosts can happen either through direct or indirect contact via fomites [1], as well as possibly by aerosols [8]. The typical clinical signs of FCV infection are painful oral and lingual ulcerations, gingivitis/stomatitis, fever, inappetence and reduced general condition, with high morbidity and low mortality [1,7,9]. However, FCV can, in rare cases, lead to virulent systemic disease whereby cats have severe mucosal and/or cutaneous ulcerations and inner organ involvement, and the mortality rates are as high as 60% [10,11,12]. Vaccines against FCV are commercially available and, in Europe, consist mainly of either modified-live single strain vaccines (FCV F9), inactivated single strain vaccines (FCV 255) or inactivated double strain vaccines (FCV G1 and FCV 431) for parenteral use. FCV vaccines protect against severe disease but they do not induce sterilising immunity and the high evolution rate, as is usual for RNA viruses, presents a challenge for efficient and broadly-acting FCV vaccines [13]. The effect of all FCV vaccines on reducing severe clinical signs has been shown in the past using various protocols of vaccination and challenge infections [14,15,16,17,18,19]. Controversial results have been published about the neutralisation potential of the FCV F9 vaccine after decades of its use in the feline population. Some studies suggest that the prolonged use of the FCV vaccine strain F9 might have driven the evolution of circulating FCV strains towards vaccine-resistant variants [20,21], whereas other studies do not confirm the divergence of field viruses from the vaccine strain F9 [22,23]. FCV-F9-like variants have been found in the feline population [24,25], but short-term oral FCV F9 shedding after parental vaccination has seldom been documented [16,26]. Furthermore, the propagation of live F9 virus in cell culture over decades might have caused viral evolution in the F9 strain itself. Usually, culturing reduces the viral quasispecies diversity [27], and in 1995, Pedersen and Hawkins [16] proposed that vaccine strains derived from FCV F9 might not be as widely cross-protective as the parental strain. In contrast, in a Swiss study, we found significant protection of cats against FCV infection (shedding) by vaccination; F9 vaccines were mainly used at that time [7]. The FCV F9 vaccine is still widely used, but to our knowledge no recent vaccination and challenge study has been performed.

The goals of the present study were: (1) to assess possible shedding of the FCV F9 vaccine virus after initial vaccination and a revaccination; (2) to examine the protective effect of the FCV F9 vaccine towards two recently collected Swiss FCV field strains in two sequential experimental infections; and (3) to investigate the effect of the FCV F9 vaccination on the duration and amount of FCV shedding, RNAemia, haematological changes and the acute phase protein reaction after experimental infections.

## 2. Materials and Methods

### 2.1. Animals

Ten male cats, eight weeks of age and from an approved specified pathogen-free (SPF) cat breeding facility, were used in this study. The results concerning environmental contamination after experimental FCV infection using these same cats have been described earlier [8]. Briefly, the cats were group-housed in a confined university facility under ethologically and hygienically ideal conditions [28]. All experiments were conducted according to Swiss law and were approved by the veterinary office of the canton of Zurich (TVB ZH095/15, issued 11 August 2015). After the arrival of the cats and prior to the start of the experiment, each cat was clinically examined, and samples were collected to confirm the cats’ SPF status, as described previously [8]. Before the start of the experiment, the cats were trained for general examination and sample collection with positive reinforcement training. All sample collections and clinical examinations were performed without anaesthesia in a stress-free manner. At the age of 23 weeks, corresponding to day 57 after the first injection of FCV Vaccination I, all cats were neutered to avoid an influence of sex hormones on the study parameters [29].

### 2.2. FCV Challenge Viruses

Both challenge viruses were field isolates and have been briefly described previously [8]. In the following, more details, particularly concerning the clinical presentation and vaccination history of the cats from which the challenge strains originated, are given.

**Figure 1:**
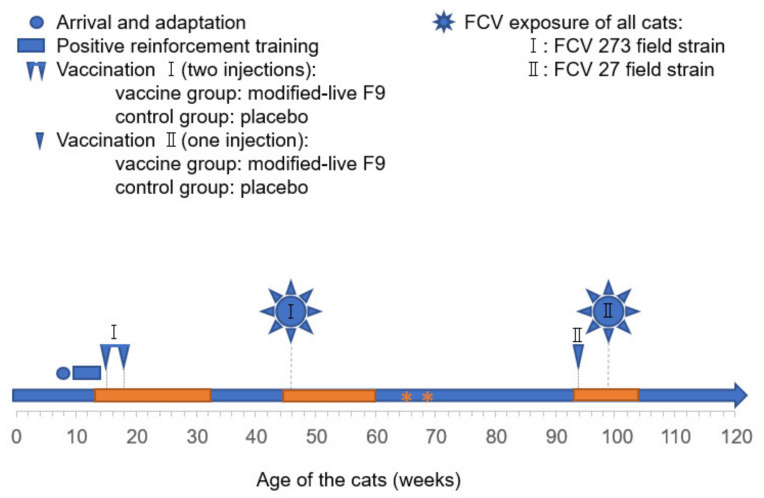
Study design (adapted from Spiri et al., 2019 [8]). The cats were adapted and trained for sample collection. At the age of 15 and 18 weeks, the cats were vaccinated twice subcutaneously (FCV F9 or placebo vaccine; FCV Vaccination I). Seven months later, at the age of 46 weeks, all cats were challenged with FCV 273 (FCV Challenge I). Eleven months later, at the age of 94 weeks, all cats were revaccinated once (FCV F9 or placebo vaccine; FCV Vaccination II) prior to the second FCV challenge (FCV Challenge II) with FCV 27 at 99 weeks of age. Cytobrush and blood samples were collected for the current study in the periods indicated by orange boxes. Two additional blood collections are indicated with orange asterisks.

The FCV 273 isolate used in FCV Challenge I (Figure 1) originated from a privately-owned domestic cat from the canton of Neuchâtel (the western part of Switzerland) and was collected in 2013 [7,8]. The nonpedigree cat was 13 years old, male, castrated, had been suffering from diabetes mellitus for several years, and had presented with chronic stomatitis/gingivitis, caudal stomatitis and lingual ulcerations for approximately one year. The cat was kept as a single cat and had no outdoor access. It was PCR-negative for feline herpesvirus (FHV)-1 and *Chlamydia felis*. The cat had received basic immunisation with Leucorifelin (Merial, Lyon, France; FCV component in the vaccine: 255) 13 years ago and was then revaccinated annually with Leucorifelin and subsequently Feligen CRP (Virbac, Switzerland; FCV component in the vaccine: F9). The cat had been receiving interferon orally for four months, starting one year before the sample collection. The cat was additionally treated with amoxicillin (Amoxicat, Biokema SA, Crissier-Lausanne, Switzerland) for two weeks, starting eight weeks before the sample collection.

The FCV 27 isolate used in FCV Challenge II (Figure 1) originated from a privately owned cat, from the canton of Schaffhausen (Northern part of Switzerland), and was collected in 2012. The Norwegian Forest cat was seven years old, female, intact, and suffering from an oral ulceration on the hard palate. The cat lived in a breeding colony with eight other cats and with outdoor access. The cat was PCR-negative for FHV-1 and *Chlamydia felis*. It had received a basic immunisation (two vaccinations three weeks apart) seven years ago and was revaccinated annually with Feligen CRP-Leucogen (Virbac, Switzerland) for five years (FCV component in the vaccine: F9). The cat had not received any treatment before the sample collection. Both challenge viruses were collected as part of a diagnostic workup [7]; no ethical approval was necessary, in compliance with Swiss regulations [30].

The neutralisation patterns of both challenge viruses towards the vaccine strain FCV F9 were tested for the current study by virus neutralisation assays, as previously described [21]. Briefly, the antisera to FCV F9 were produced by infecting SPF cats with the corresponding vaccine strains [21]. From each SPF cat serum, six serial dilutions were prepared, starting at 1:5 and continuing with 3-fold dilutions (1:5, 1:15, 1:45, 1:135, 1:405, 1:1215). Fifty microliters of each serum dilution were transferred in quadruplicate to a 96-well plate. FCV strains were diluted to 100 50% tissue culture infective dose (TCID_50_)/mL with serum-free 1 × Dulbecco’s Modified Eagle’s Medium (DMEM; Gibco, Paisley, UK), supplemented with 10% heat-inactivated foetal bovine serum (Gibco), 1% L-glutamine 200 mM (Gibco), 2.4% penicillin 10,000 U/mL and streptomycin 10,000 µg/mL (Gibco), 1% sodium pyruvate 100 mM (Gibco), and 1% gentamicin (10 mg/mL) (Gibco). Fifty microliters of 100 TCID_50_/mL of each virus and 50 µL of each serum dilution were incubated for 2 h at 37 °C so the antibodies could neutralise the virus. After 2 h, 3 × 10^4^ feline embryonic A cells in 150 µL of DMEM complete were added to each well. After 48 h of incubation at 37 °C, each well was assessed for cytopathic effect (CPE). The titre is the reciprocal of the serum dilution where 50% or more of the wells show a CPE. Positive (virus and cells only) and negative (cells only) controls were run in parallel on the same plate. Both challenge viruses were similarly neutralised by two sera raised against FCV F9 (Table 1). The FCV F9 homologous titres were 135 and 405 for serum obtained from cat 1 (S1) and cat 2 (S2), respectively. The sequence identity between FCV 27 and FCV 273 was 77.4% and the sequence identity to the vaccine strain FCV F9 was comparable for both challenge viruses (FCV 27 75.8%; FCV 273 74.7%).

### 2.3. FCV Vaccinations and FCV Experimental Infections

The study design has been described previously [8]. Briefly, 10 cats were randomly assigned to the vaccine (Cat ID: JJG4, JJG6, JJH3, JJI1, JJI2) or the control group (Cat ID: JJF1, JJG3, JJH2, JJI3, JJI4). The five cats in the vaccine group were vaccinated subcutaneously into the lateral abdominal wall at 15 and 18 weeks of age (Figure 1; FCV Vaccination I) with a commercially available modified-live virus vaccine containing FCV F9, feline herpesvirus and feline panleukopeniavirus (Feligen^®^ CRP, Virbac AG, Glattbrugg, Switzerland). The five cats in the control group were injected only with the sterile water that is used to reconstitute the lyophilised vaccine. All vaccinations and the challenge infections were performed by an experienced senior veterinarian and all cats were vaccinated or placebo injected consecutively within one hour and all cats were challenged consecutively within one hour. After subcutaneous vaccinations, the fur of the cats was checked for accidental spillage or leakage of vaccine material. All cats were infected oronasally with FCV 273 (FCV Challenge I; 1.5 × 10^6^ TCID_50_/mL), seven months after the second injection of the first vaccination or placebo vaccination (Figure 1; FCV Vaccination I). The cats were revaccinated once subcutaneously into the lateral abdominal wall with Feligen^®^ CRP (Virbac AG) or the placebo, respectively, 11 months after FCV Challenge I (Figure 1; FCV Vaccination II). One month after revaccination (Vaccination II), all cats were oronasally infected with FCV 27 (3.2 × 10^6^ TCID_50_/mL) (FCV Challenge II).

### 2.4. Assessment of Clinical Signs

A clinical score sheet was used to assess clinical signs (Table 2) [31]. Depending on the severity, the duration and the starting point of clinical signs, scores were assigned. Sneezing, coughing and open-mouthed breathing were assessed first thing in the morning, prior to handling the cats. Rectal body temperature was obtained before blood collection to avoid increased body temperatures due to excitement. The cats were monitored for clinical signs as described in the clinical score sheet and the following parameters were added to the assessment: gingivitis, stomatitis, peripheral lymph node enlargement, decreased body weight and decreased general condition. The clinical signs were assessed before and after FCV Vaccination I and II and before and after FCV Challenge I and II, as shown in Table 3. The clinical signs, assessed daily for a 15-day period after FCV Challenges I and II according to the score sheet, were used to assess the total and maximum score. Scores from each day during the 15-day observation period were added and a total score for each cat was calculated. The maximum score represented the highest score reached in every cat during the 15-day observation period. Two trained and experienced veterinarians performed the clinical scoring. These two veterinarians and all the caretakers involved in cat handling were blinded towards the vaccination statuses of the cats. Cats with increased body temperature that showed apathy and anorexia received the anti-inflammatory drug meloxicam (Metacam^®^ 0.5 mg/mL, Boehringer Ingelheim, Basel, Switzerland): an initial dose of 0.1 mg/kg meloxicam SID orally for one day and subsequently 0.05 mg/kg SID orally for two consecutive days.

### 2.5. Oropharyngeal Cytobrush Sample Collection from Cats

Cytobrush samples were collected by rolling endocervical sampling brushes (Deltalabs S. L. U., Barcelona, Spain) over the hard palate and the tongue of the cat. The collection time points of cytobrush samples for virus isolation on Crandell–Rees feline kidney (CRFK) cell culture is described in Spiri et al., 2019 [8]. For nucleic acid extraction, the cytobrush samples were directly transferred to 1.5-mL microtubes (Sarstedt AG & Co. KG, Nümbrecht, Germany) containing 300 μL DNA/RNA shield (Zymo Research Europe GmbH, Freiburg i. B., Germany) and stored at –80 °C until further use. The time points of sample collection are indicated in Figure 1 and detailed in Table 3.

### 2.6. Blood Collection, Processing, and Analyses

Blood samples were collected from the *vena cephalica*. Ethylenediaminetetraacetic acid (EDTA) anticoagulated blood was used for haematology and total nucleic acid (TNA) extraction. Complete blood count and leukocyte differentiation, consisting of segmented and banded neutrophils, lymphocytes, monocytes, eosinophils and basophils, was performed with a Sysmex XT2000iV (Sysmex, Kobe, Japan) validated for feline blood samples [32]. From each sample, a blood smear was prepared, stained with Wright–Giemsa stain, and the differentiation result from the SysmexXT2000iV was verified by a visual evaluation using a microscope. If visual verification failed or banded neutrophils were present, a manual leukocyte differentiation was performed. Additionally, cell morphology was assessed in the blood smears. The laboratory’s own established and haematology analyser-specific reference ranges for adult cats were used from FCV Challenge I on when cats were 11 months old [32]. Whole native blood was centrifuged at 1862×g for 5 min; serum was collected and one aliquot was directly analysed for acute phase protein serum amyloid A (SAA) using a latex agglutination turbidimetric immunoassay reaction (LZ Test “Eiken” SAA, Eiken Chemical Co., Ltd., Tokyo, Japan) on a Cobas C501 clinical chemistry analyser (Roche Diagnostics AG, Rotkreuz, Switzerland) [33].

### 2.7. Nucleic Acid Extraction and PCR

Prior to TNA extraction, oropharyngeal cytobrush samples stored in DNA/RNA shield (Zymo Research) were thoroughly mixed and incubated at 40 °C for 10 min. TNA was extracted from 100 µL EDTA anticoagulated blood or 200 µL RNA/DNA shield (Zymo Research) using the MagNa Pure LC (Roche Diagnostics AG) and the MagNa Pure LC Total Nucleic Acid Isolation Kit (Roche Diagnostics AG) according to the manufacturer’s instructions. Positive and negative RT-qPCR controls were run in parallel. Two different real-time RT-qPCR assays (FCV RT-qPCR S1 and S2) were used to detect FCV, as previously described [7]. For a semiquantitative analysis of FCV viral loads in cytobrush and blood samples, FCV RNA standards were run in parallel.

### 2.8. Statistics

All data were compiled in Microsoft^®^ Excel^®^ 2016 for Microsoft 365, version 2103 and analysed using GraphPad Prism 8 software (La Jolla CA, USA). Fisher’s exact test was used to test for differences in proportions. The Mann–Whitney U test was used to test for differences between the vaccine and the control group. Friedman’s test and Dunn’s post-test tested the changes over time within a group.

## 3. Results

### 3.1. Outcome of the First FCV Vaccination (FCV Vaccination I)

**Figure 2:**
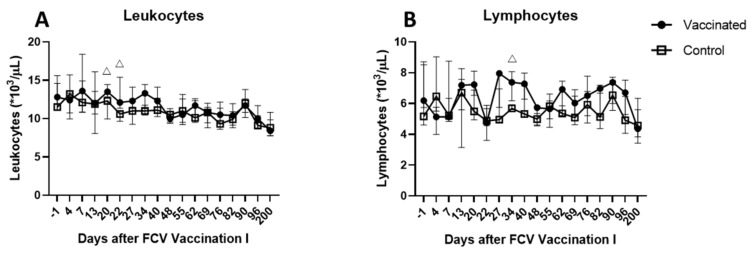
Leukocyte and lymphocyte counts after FCV Vaccination I. Median (± interquartile range (IQR)) absolute cell count of leukocytes (**A**) and lymphocytes (**B**) after FCV Vaccination I in the vaccine and the control group. Statistically significant differences between the control and the vaccine group are indicated with an open triangle; △ denotes significantly higher in the vaccine group P_MWU_ ≤ 0.05. Days after FCV Vaccination I on the *x*-axis indicate the days after the first injection of FCV Vaccination I. The second injection of FCV Vaccination I was performed on day21.

No clinical signs were observed and no FCV shedding was detected in any of the cats after neither injection of FCV Vaccination I using virus isolation or RT-qPCR. No leakage or spilling of vaccine material to the fur of the cats was detected. Moreover, no viral RNA was detectable in the blood, as tested by RT-qPCR. There was no significant difference in rectal body temperature between the two groups at any of the time points tested (P_MWU_ > 0.05). The leukocyte count was significantly higher in the vaccinated animals than in the control animals on day 20 after the first injection of FCV Vaccination I (P_MWU_ = 0.0079) and on day 1 after the second injection of FCV Vaccination I (P_MWU_ = 0.0317) (Figure 2a). The lymphocyte count was significantly higher in the vaccinated animals on day 13 after the second injection of FCV Vaccination I (P_MWU_ = 0.0317) (Figure 2b). All other haematological parameters (total neutrophils, banded neutrophils, monocytes, eosinophils and basophils) were not significantly different between the groups.

### 3.2. Outcome of the First Heterologous FCV Infection (FCV Challenge I)

**Figure 3:**
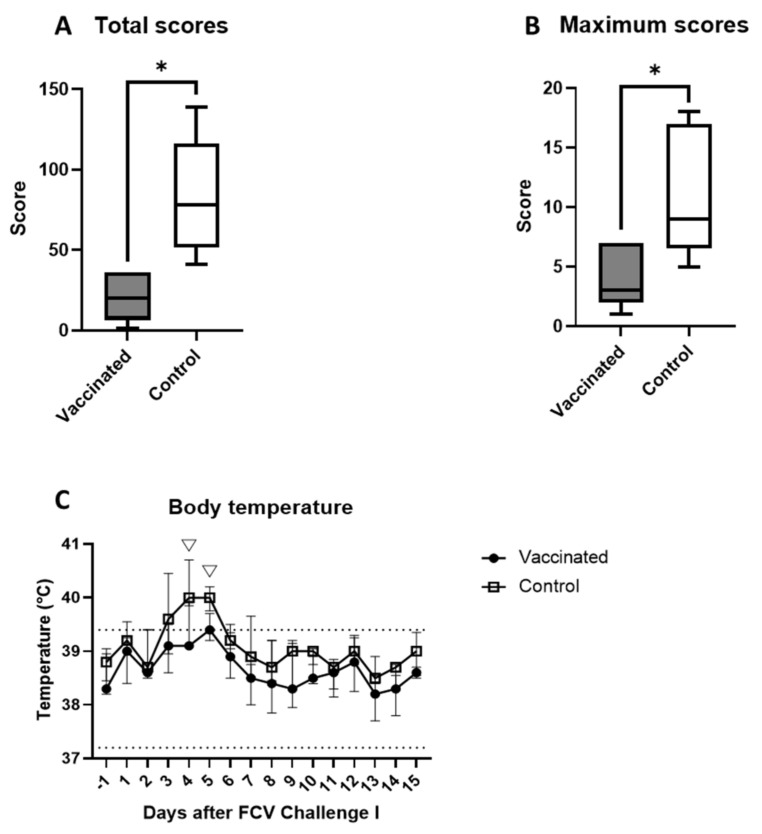
Clinical scoring and body temperature after FCV Challenge I. Box and whisker plots of total (**A**) and maximum scores (**B**) after FCV Challenge I, * P_MWU_ ≤ 0.05. The line inside the box shows the median, and the lower and upper borders of the box indicate the 25th and 75th percentile, respectively. Whiskers represent the minimum and maximum values. Median ± IQR of the body temperature (**C**) after FCV Challenge I; statistically significant differences between the control and the vaccine group in C are indicated with an open triangle; ▽ denotes significantly lower in the vaccine group P_MWU_ ≤ 0.05. The lower and upper limit of the reference range of normal body temperature is indicated by two dotted lines. Three cats in the control group received anti-inflammatory treatment starting at days 3–5 after FCV Challenge I.

All cats showed some clinical signs after the first experimental infection (FCV 273). Clinical signs consisted mainly of oral ulcerations (day 9: 2/5 vaccinated cats and 5/5 control cats), fever (day 4 and 5: 2/5 vaccinated cats and 5/5 control cats), enlarged lymph nodes (days 6–3: 5/5 vaccinated cats and 5/5 control cats) and reduced general condition (day 5: 0/5 vaccinated cats and 4/5 control cats). No sneezing, dyspnoea, open-mouthed breathing or rhinitis was observed in any of the cats, and none of the cats died during the observation period. The total scores (P_MWU_ = 0.0079) and maximum scores (P_MWU_ = 0.0317) were significantly lower in the vaccinated group than in the control group (Figure 3a,b). Oral ulcerations started to be present between day 5 and day 9 and were detectable up to day 22 in most cats, except for cat JJI4 of the control group; oral ulcerations were present up to day 52 in this cat. Three cats (JJH3, JJI1 and JJI2) from the vaccine group never showed oral ulcerations. After day 15, the presence of oral ulcerations was only assessed twice a week and therefore the exact duration of the presence of oral ulcerations could not be determined and no statistical assessment was performed. A reduced general condition (reduced physical activity, lower appetite, reduced grooming and increased resting behaviour) was present in six cats for a minimum of one day up to a maximum of six days. In three cats of the vaccine group (JJG4, JJH3, and JJI1) and one cat of the control group (JJH2), a reduced general condition was never detected. The duration of reduced general condition was not significantly different between the groups. Enlarged mandibular and/or popliteal lymph nodes were present in all cats and the enlargement was detectable up to day 108 after FCV Challenge I. No statistical assessment was performed on the duration of enlarged lymph nodes.

The rectal body temperature was significantly higher in the control group than in the vaccinated group on day 4 (P_MWU_ = 0.0476) and day 5 (P_MWU_ = 0.0238) after FCV Challenge I (Figure 3c). This was in part due to an increased temperature of up to 40.3 °C in cats JJG3, JJI3, and JJI4 of the control group; the increased body temperature was accompanied by apathy and anorexia in these cats. For animal welfare reasons, the three cats received an anti-inflammatory drug (see Section 2.4) starting on day 3, 4 and 5, respectively, after experimental infection, and the body temperature was not significantly different thereafter (Figure 3c). The duration of fever in all the cats was 1–6 days and was not significantly different between the vaccinated and the control animals. Significant differences in terms of the changes over time of the body temperature in each group are shown in Table A1.

All 10 cats shed virus after FCV Challenge I and the duration and frequency analysed by virus isolation was not significantly different between the vaccinated and the control group as described previously [8]. Briefly, all cats were shedding the replicating virus until at least day 9 after FCV Challenge I. Most cats ceased FCV shedding between days 9 and 15 after FCV Challenge I. One cat (JJH3) from the vaccine group ceased shedding only between days 63 and 71 after FCV Challenge I.

**Figure 4:**
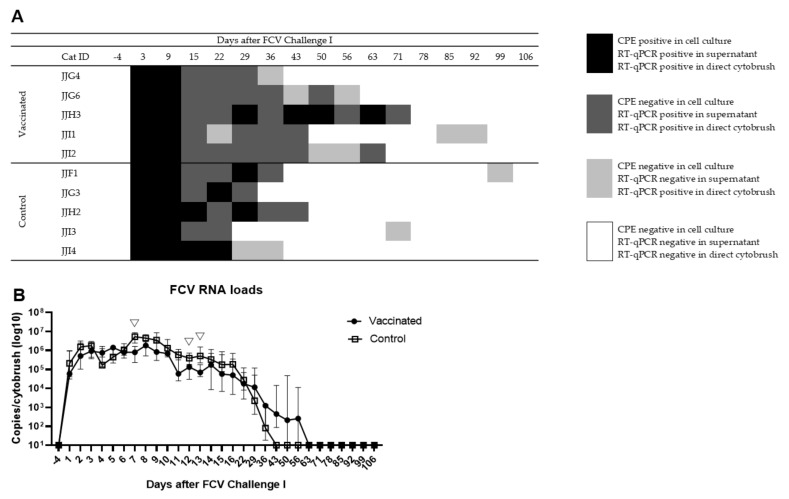
FCV shedding from the oropharynx of vaccinated and control cats after FCV Challenge I. FCV shedding detection by cell culture and RT-qPCR (**A**) and FCV RNA loads (median ± IQR) in oropharyngeal cytobrush samples after FCV Challenge I (**B**). Statistically significant differences between the vaccine and the control group are indicated with an open triangle; ▽ denotes significantly lower in the vaccine group P_MWU_ ≤ 0.05.

For the present study, viral RNA shedding and RNA loads were also determined by RT-qPCR directly from oropharyngeal cytobrush samples without prior cell culture. The results of shedding of replication-competent FCV tested by cell culture using CRFK cells have been shown previously [8]. The combined results of virus isolation, RT-qPCR results of the cell culture supernatant, RT-qPCR from direct cytobrushes and the FCV RNA loads are shown in Figure 4. All cats were positive in RT-qPCR of direct cytobrushes on days 1–16 and day 22 after FCV Challenge I (Figure 4a,b). The duration and frequency of cats shedding FCV RNA detected by direct cytobrushes were not significantly different between the vaccinated and the control animals at any timepoint.

Viral RNA was detectable in the cytobrushes as early as on day 1 after FCV Challenge I in all 10 cats. The viral RNA load in the oropharyngeal cytobrushes was up to 12.5 × 10^6^ copies/cytobrush (cat JJH2 of the control group, day 7) after FCV Challenge I (Figure 4b). The viral RNA loads then decreased over the time course of the infection (Figure 4b), and the last FCV positive result was found on day 99 in a cat from the control group (JJF1; Figure 4a). Vaccinated cats had significantly lower FCV RNA loads on days 7 (P_MWU_ = 0.0159), 12 (P_MWU_ = 0.0159), and 13 (P_MWU_ = 0.0317) compared to control cats (Figure 4b).

**Figure 5:**
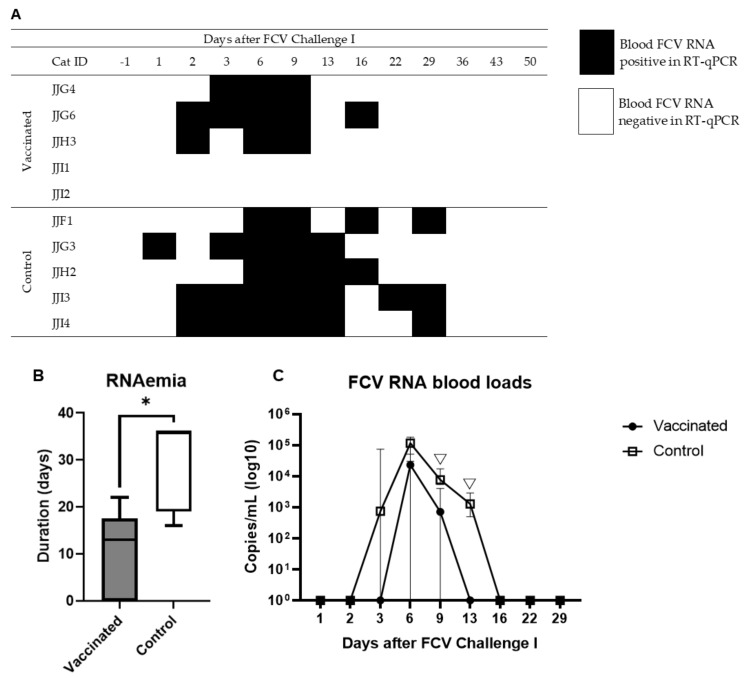
RNAemia (detection of viral RNA by RT-qPCR in peripheral blood) after FCV Challenge I. RNAemia in blood of vaccinated and control cats (**A**); box and whisker plots of the duration of RNAemia, the line inside the box shows the median and the lower and upper borders of the box indicate the 25th and 75th percentile, respectively; whiskers represent the minimum and maximum values, * P_MWU_ ≤ 0.05 (**B**); FCV RNA loads (median ± IQR) in blood (**C**); statistically significant differences between the vaccine group and the control are indicated with an open triangle; ▽ denotes significantly lower in the vaccine group, P_MWU_ ≤ 0.05.

RNAemia (detection of viral RNA in peripheral EDTA anticoagulated whole blood determined by RT-qPCR) was detected in 8/10 cats from day 1 to day 29 after FCV Challenge I (Figure 5a). The median duration of RNAemia in samples from the vaccinated cats was 13 days (IQR ± 17.5, Min: 0, Max: 22) and 36 days in samples from the control cats (IQR ± 17, Min: 16, Max: 36); the duration of RNAemia was significantly shorter in the vaccinated than in the control animals (P_MWU_ = 0.0238) (Figure 5b). Two cats (JJI1 and JJI2) from the vaccine group never showed RNAemia (no detectable viral RNA in the blood of these cats at any timepoint tested) (Figure 5a). On day 13 after FCV Challenge I, the proportion of cats having FCV RNAemia was significantly different between the groups (P_F_ = 0.0476), with no cat having RNAemia in the vaccine group and 4/5 cats having RNAemia in the control group. FCV blood RNA loads were significantly lower in the vaccine group on day 9 (P_MWU_ = 0.0317) and 13 (P_MWU_ = 0.0467) after FCV Challenge I compared to the control group (Figure 5c). Maximum blood RNA loads of 185,423 copies/mL blood were found in cat JJF1 of the control group on day 6 after FCV Challenge I.

**Figure 6:**
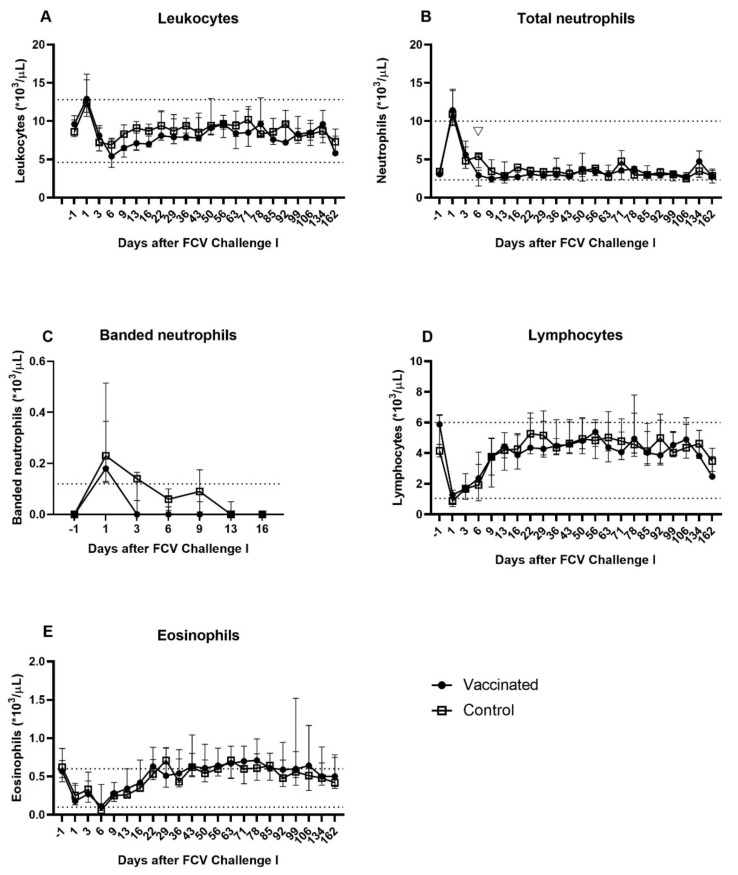
Absolute total and differential leukocyte counts after FCV Challenge I. Median (± IQR) absolute cell count of leukocytes (**A**), total (segmented and banded) neutrophils (**B**), banded neutrophils (**C**), lymphocytes (**D**), and eosinophils (**E**) after FCV Challenge I. The area between the dotted lines indicates the reference range. Statistically significant differences between the control and the vaccine group in B are indicated with an open triangle;▽ denotes significantly lower in the vaccine group, P_MWU_ ≤ 0.05.

All cats underwent a complete blood cell count after FCV Challenge I. The leukocyte count increased in 9/10 cats on day 1 after the first challenge and mild to moderate leukocytosis (12.9–18.8 × 10^3^/µL) was seen in 2/5 control cats (JJH2 and JJI3) and 3/5 vaccinated cats (JJG4, JJI1, and JJI2) (Figure 6a). On day 6, mild leukopenia was detected in two cats of the vaccine group (JJG4: 4.5 × 10^3^ leukocytes/µL and JJG6: 3.4 × 10^3^ leukocytes/µL) and on day 9 in one cat of the control group (JJG3: 4.1 × 10^3^ leukocytes/µL) (Figure 6a). All leukocyte counts were within the reference range from day 13 until day 162 (Figure 6a). All cats showed a transient increase in the total neutrophil (banded and segmented) count after FCV Challenge I and 4/5 vaccinated cats (JJG4, JJH3, JJI1, and JJI2) and 3/5 control cats (JJH2, JJI3, and JJI4) had mild to moderate neutrophilia (10.66–15.75 × 10^3^ neutrophils/µL) on day 1 after FCV Challenge I (Figure 6b). A very mild left shift was detected on day 1 after FCV Challenge I in both groups (Figure 6c). The banded neutrophil count was back into the reference range on day 3 in all vaccinated animals and on day 6 in all control animals (Figure 6c). A mild neutropenia was detected in vaccinated and control animals from day 6 to 16 after FCV Challenge I (Figure 6b). The total neutrophil count on day 6 after FCV Challenge I was significantly lower in the vaccinated cats than in the control cats (P_MWU_ = 0.0159) (Figure 6b). A transient mild to moderate lymphopenia was seen in some cats of both groups from day 1 to 6 after FCV Challenge I (Figure 6d). A transient decrease in the eosinophilic count was detected in all cats after FCV Challenge I and mild eosinopenia was seen in one cat of the vaccine group on day 3, and in two cats of the vaccine group and in four cats of the control group on day 6 after FCV Challenge I (Figure 6e). By day 9, the eosinophil count was back to the reference range in all cats. No values outside the reference range were detected for basophils. There were no significant differences between the groups regarding banded neutrophils, lymphocytes, eosinophils, monocytes and basophils. Significant differences in changes over time of the leukocyte differentiation in each group are shown in the Appendix A (Table A1).

**Figure 7:**
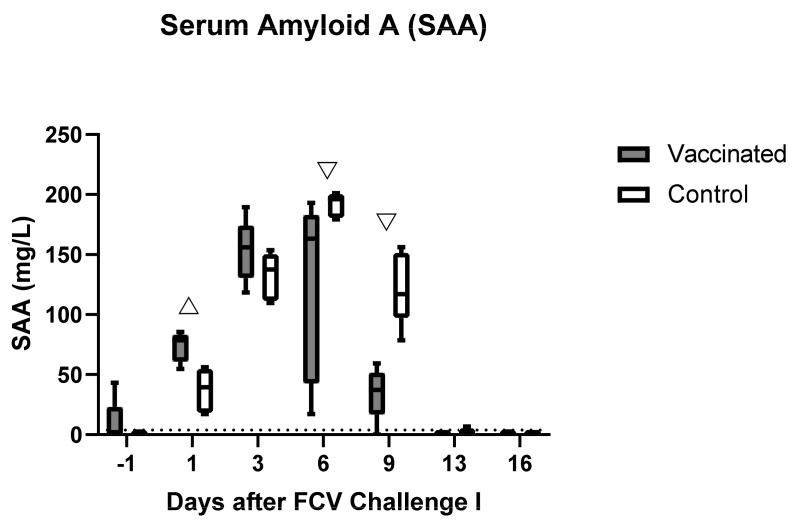
Box and whisker plots of the acute phase protein SAA after FCV Challenge I. The line inside the box shows the median and the lower and upper border of the box indicates the 25th and 75th percentile, respectively. Whiskers represent minimum and maximum values. The dotted line indicates the upper limit of the reference range. Significant differences between the vaccinated group and the control group are indicated with an open triangle; ▽ denotes significantly lower in the vaccinated group, P_MWU_ ≤ 0.05; △ denotes significantly higher in the vaccinated group, P_MWU_ ≤ 0.05.

A transient increase in SAA was observed in both groups after FCV Challenge I, with the highest value of 201.1 mg/L in cat JJH2 of the control group on day 6 and a constant decrease in all cats afterwards. By day 16, all SAA values were back to normal (Figure 7). The concentration of SAA was significantly higher in the vaccinated animals on day 1 after the FCV Challenge I (P_MWU_ = 0.0159). However, on days 6 and 9 the SAA concentration was higher in the control cats (P_MWU_ = 0.0317 and P_MWU_ = 0.0079). Cat JJG6 from the vaccine group had a preinfectional SAA value above the reference range of 43.2 mg/L. Significant differences in the changes over time in each group of SAA are shown in the Appendix A (Table A1).

### 3.3. Outcome of the Revaccination (FCV Vaccination II)

No spilling or leakage of vaccine material was detected on the fur of the cats. No clinical signs were observed in any of the cats after revaccination (vaccine or placebo). None of the cats showed increased body temperature. No FCV shedding from the oropharynx and no viral RNA in blood was detected by RT-qPCR after FCV Vaccination II. All haematological parameters remained within the reference range.

### 3.4. Outcome of the Second Heterologous FCV Infection (FCV Challenge II)

The second FCV challenge was performed one year after FCV Challenge I and one month after FCV Vaccination II with the heterologous FCV field isolate, FCV 27. Besides a mild increase in rectal body temperature and mildly enlarged popliteal and mandibular lymph nodes in some cats, no clinical signs were observed after FCV Challenge II. Cat JJF1 from the control group, showed rectal temperatures >39.4 °C before the second challenge and on 8 out of 15 days of the daily observation period after the second challenge. Due to the absence of clinical signs recorded in the score sheet, except for increased body temperature, no clinical scores were calculated after the second challenge.

**Figure 8:**
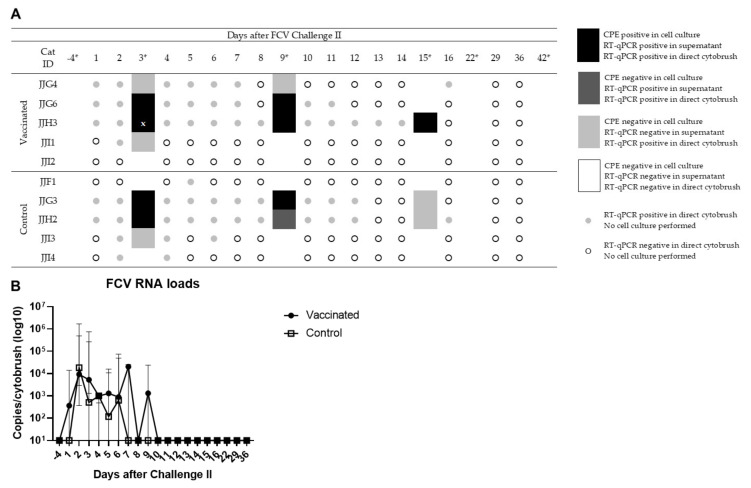
FCV shedding from the oropharynx of vaccinated and control cats after FCV Challenge II. FCV shedding was detected by cell culture, by RT-qPCR of cell culture supernatants and by RT-qPCR of direct cytobrushes. Sampling days with cell culture are indicated with an asterisk *; x describes a missing value of the RT-qPCR result of the direct cytobrush (**A**). Median (±IQR) of FCV RNA loads in oropharyngeal cytobrush samples collected after FCV Challenge II (**B**).

The results of the FCV shedding, as determined by virus isolation, have been described previously [8]. Briefly, 2/5 vaccinated and 2/5 control cats were shedding FCV on day 3. Three cats stopped shedding by day 15; cat JJH3 had the longest shedding period and stopped by day 22. Cats JJF1 and JJI2 never tested FCV-positive in a cell culture. The combined results of virus isolation, RT-qPCR results of cell culture supernatants, RT-qPCR from direct cytobrushes and the FCV RNA loads are shown in Figure 8a,b). FCV shedding measured in direct oropharyngeal cytobrushes by RT-qPCR was observed in 9/10; cat JJI2 stayed FCV-negative throughout the time course of infection (Figure 8a). FCV loads were much lower than after the first FCV challenge: maximal viral RNA loads with 2.4 × 10^6^ copies/cytobrush were detected in cat JJH2 of the control group on day 2 after FCV Challenge II. Viral RNA loads were not significantly different between the two groups (Figure 8b). No data of oropharyngeal virus shedding detected by RT-qPCR and FCV RNA loads were available from cat JJH3 on day 3 after FCV Challenge II due to a lack of sufficient sample material (Figure 8a). No FCV RNA was detected in the blood of any cat after FCV Challenge II.

**Figure 9:**
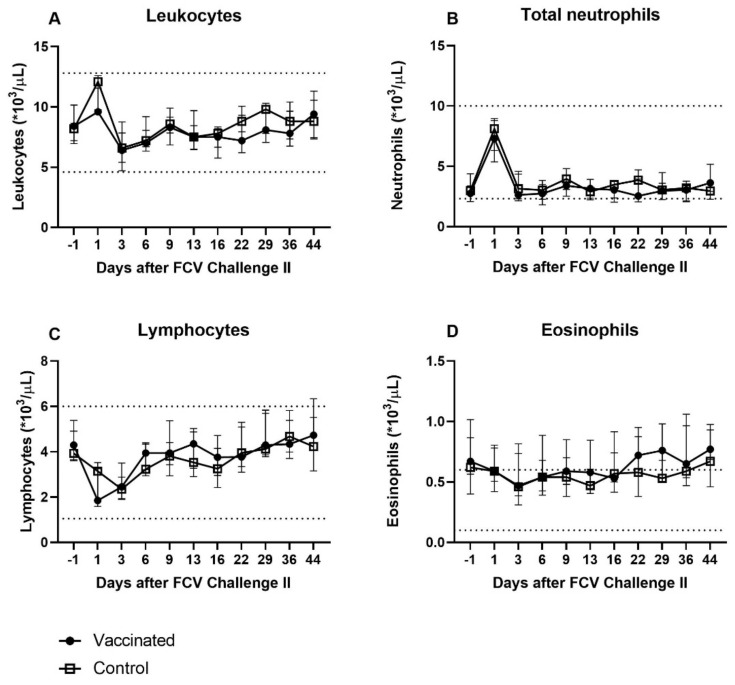
Absolute total and differential leukocyte counts after FCV Challenge II. Median (± IQR) absolute cell count of leukocytes (**A**), total (segmented) neutrophils (**B**), lymphocytes (**C**), and eosinophils (**D**) after FCV Challenge II in the vaccine group and the control group. The lower and upper limits of the reference range are indicated by the dotted lines.

As after FCV Challenge I, a transient increase in leukocytes and total (segmented) neutrophils was observed on day 1 after FCV Challenge II in both groups, but less pronounced compared to FCV Challenge I (Figure 9a,b). No banded neutrophils were detected after FCV Challenge II. A transient decrease in lymphocytes and eosinophils was also seen on days 1 and 3 after FCV Challenge II in both groups (Figure 9c,d). No changes were detected in monocytes and basophil counts. Except for mild eosinophilia in the vaccinated cats from day 22 to day 44 after FCV Challenge II, all median haematological values were within the reference range and no statistically significant differences were seen between the two groups (Figure 9a–d). Cat JJI2 of the vaccine group showed mild eosinophilia (1.29 × 10^3^/µL) already before FCV Challenge II and constantly afterwards throughout the whole observation period until day 44 (0.9–1.22 × 10^3^/µL). Significant differences in the changes over time in each group are shown in Table A2.

**Figure 10:**
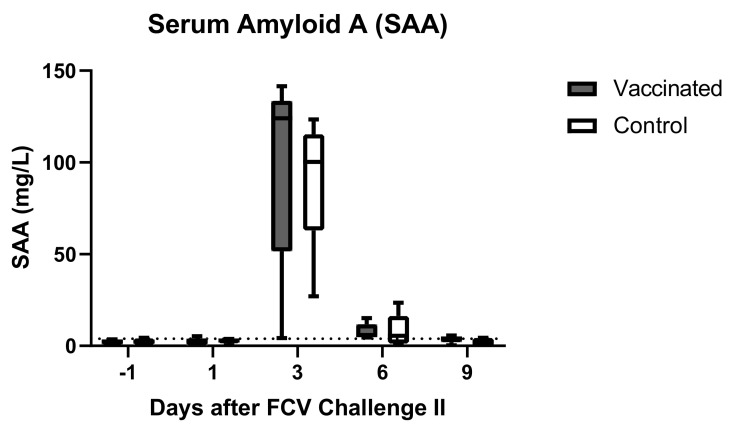
Acute phase protein reaction after FCV Challenge II. Box and whisker plots of the acute phase protein SAA after FCV Challenge II. The line inside the box shows the median and the lower and upper borders of the box indicate the 25th and 75th percentile, respectively. Whiskers represent minimum and maximum values. The dotted line indicates the upper limit of the reference range.

The acute phase protein SAA increased moderately in 9/10 cats on day 3 after FCV Challenge II (Figure 10). The maximum value was reached in cat JJI1 of the vaccine group on day 3 with 141.6 mg/L. SAA values normalised within the reference range on day 9. Cat JJI2 of the vaccine group stayed at a low level (≤4.6 mg/L) during the entire measurement period of SAA (Figure 10). This time, there were no statistically significant differences found between the two groups. Significant differences in the changes over time of SAA in each group are shown in Table A2.

## 4. Discussion

The protective effect of the FCV F9 vaccination to induce seroconversion and to reduce severe clinical signs was demonstrated in studies from the 1970s–90s using different protocols of vaccination and challenge [14,15,16,34,35]. The challenge infection by aerosolisation, used in most of these studies, did not reflect the natural method of infection and clinical signs were often located in the lower respiratory tract. Oronasal inoculation, as used in more recent studies and the present study, seems to mimic more closely the natural route of infection to induce classical FCV-related clinical signs [1].

FCV F9 has been cultivated by manufacturers to supply the vaccine production chain. Cultivation can cause minor viral evolution [27] and the current FCV F9 isolate might not be identical to the isolate obtained in the 1960s, when F9 was first described as a vaccine strain [16]. Taking this into account, to perform a FCV F9 vaccination and challenge study with the current F9 vaccine and with recently collected FCV field strains gives important insight into the present situation. The study setting, consisting of a primary FCV vaccination followed by a FCV challenge, a revaccination and a rechallenge, was chosen to mirror the situation in the field, where young and FCV-naïve cats are vaccinated and encounter different FCV variants over the course of their lives.

In the current study, SPF cats were vaccinated against FCV with a modified-live vaccine containing FCV F9 or with a placebo vaccine before they underwent two subsequent oronasal FCV challenge infections with current FCV field isolates. A protective effect of the FCV F9 vaccine was observed against the development of severe clinical signs. Furthermore, vaccinated cats had lower viral RNA loads in cytobrushes and blood, shorter duration of RNAemia and a less pronounced acute phase protein reaction than the control cats. Our study documented for the first time the course of RNAemia, of haematological parameters, and of the acute phase protein SAA after an experimental FCV infection with a nonvirulent-systemic virus.

Neither after the initial FCV vaccination of naïve cats nor after revaccination 1.5 years later was any shedding of vaccine virus from the oropharyngeal region or RNAemia detected in any of the cats. There are reports of oral shedding after modified-live FCV vaccination [16,26,36] and FCV F9-like variants have been detected in phylogenetic analyses of FCV isolates originating from various countries such as the UK [25] and Switzerland [24]. After the application of intranasal modified-live FCV vaccines, mild and transient clinical signs and viral shedding are possible [16]. An accidental oral administration of a subcutaneous modified-live vaccine or inadvertently spilled vaccine material on the fur of the cat and subsequent grooming can lead to oropharyngeal uptake of the pathogen and local replication and mild clinical signs are possible [16]. The presence of live and replication-competent vaccine virus in a susceptible population could favour the virus’s evolution towards immune escape variants [20]. Therefore, vaccine virus shedding is not desirable. The most recent FCV vaccine on the European market consists of two inactivated FCV strains [17,37]. Pathogens in inactivated vaccines are unable to replicate and therefore cannot induce clinical signs of the infection [38]. However, it is generally thought that inactivated vaccines cause a different immune response, which is directed more to the humoral pathway, and which usually needs enhancement by an adjuvant [38]. For FCV, cellular immunity also seems to play a role in vaccine protection [39,40,41], as cats without detectable antibodies can be protected from disease [37,42]. In the present study, accidental leakage or spilling onto the fur of the cats was not detected and an oral uptake of vaccine virus was therefore unlikely. This might be one of the reasons for the absence of FCV oropharyngeal shedding after vaccination.

Subcutaneously applied modified-live vaccines replicate within the patient to elicit an immune response. Viremia or RNAemia/DNAemia after modified-live vaccination has been demonstrated for some pathogens in animals [43] or humans [44], but data on RNAemia in FCV-vaccinated cats are missing. For canine parvovirus, a DNA virus causing severe acute haemorrhagic gastroenteritis in dogs, viremia was detected up to 24 days after the administration of a modified-live parvovirus vaccination [45]. In immunocompromised cats, i.e., FIV-infected, the possibility of FCV vaccine virus shedding might be higher after vaccination. It has been shown that higher amounts of FCV were shed in the saliva of FIV-infected cats than in FIV-negative cats after FCV infection [46]. On the contrary, iatrogenic immunosuppression through medication did not result in lower FCV antibody titres after vaccination compared to nonimmunosuppressed cats [47]. Immunosuppression due to infectious diseases or medications can be ruled out in the present study since we were using regularly monitored SPF cats in a barrier facility and no immunosuppressive medications were given during the whole study period.

One limitation of the present study is that we used five vaccinated cats; thus, the sample size was low and FCV shedding or RNAemia after vaccination might have been detected in a low number of cats if the sample size was much larger. Additionally, the viral RNA shedding could have been below the detection limit of the RT-qPCR but, as no CPE was detected in cell culture, the presence of replication-competent FCV in these samples seems very unlikely. Furthermore, the timeframe of oropharyngeal shedding can be limited but, because we used daily sample collection in the present study, there was only a small chance of missing significant shedding of vaccine virus. To sum up, proper handling and usage of modified-live FCV virus vaccines in immunocompetent cats are recommended to prevent vaccine virus shedding and avoid further distribution of the virus vaccine strain in the feline population.

All 10 cats—five that received the FCV F9 vaccine and five that received a placebo vaccine—underwent a first oronasal challenge infection (FCV Challenge I) with a current FCV field isolate (FCV 273). All 10 cats showed FCV typical clinical signs after this infection, independent of their vaccination status. However, vaccinated cats had significantly lower maximum and total clinical scores and lower rectal body temperatures on days 4 and 5 after FCV Challenge I, indicating the protective effect of the vaccination. For animal welfare reasons, short-term nonsteroidal anti-inflammatory therapy had to be applied to some cats of the control group in the early phase of FCV Challenge I. This might have caused a confounding of the body temperature and the general condition and the differences between the groups might have been more pronounced without treatment. The healing of oral ulcerations occurred within three weeks in most cats according to the current literature [48]. However, cat JJI4 from the control group had a longer healing period, which confirmed reports from previous studies that healing can take significantly longer in individual animals [9]. Popliteal and/or mandibular lymph nodes were back to their normal size by day 85–108 after FCV Challenge I. The enlargement was present for much longer than the shedding of replication-competent FCV in most cats. Most probably, the lymph node enlargement represents the activation of the immune system because of a continuous exposition to FCV RNA, even though this RNA did not belong to replication-competent viral particles. The last RT-qPCR positive cytobrush was found on day 99, and FCV RNA was still detectable in the cat facility on day 106 after FCV Challenge I [8].

All 10 cats were shedding replication-competent virus until at least day 9 after FCV Challenge I. The duration and frequency of replication-competent FCV shedding were not significantly different between the groups, but the viral RNA loads shed from the oropharynx were significantly lower in the vaccinated cats compared to the unvaccinated controls. The impact of vaccination on the duration and extent of shedding has been controversial. Some studies have documented a reduction in the extent of shedding in vaccinated animals, but these studies did not use FCV F9 as a vaccine strain [17,18,19,49]. One study using the FCV F9 vaccine strain could not document a reduction of shedding [16], and another study detected an extension of the shedding period [50]. A one-time detection of viral RNA in a specimen does not imply the presence of replication-competent live virus. In the present study, viral RNA results were compared to oropharyngeal shedding of replication-competent FCV, as determined by cell culture. Most cats ceased shedding of replication-competent FCV between days 9 and 36 after the first experimental infection, but most cell culture supernatants were RT-qPCR positive for samples collected several weeks after that. This finding indicates that positive RT-qPCR results, but not replication-competent virus can be found in cats recovering from FCV infection. Therefore, the FCV RT-qPCR results obtained from cats recovering from a FCV infection need to be interpreted bearing the disease history in mind; positive RT-qPCR results can be found up to 70 days after cessation of shedding replication-competent virus. As a limitation, a determination of viral RNA loads from the oropharynx is not absolutely accurate and a sample-to-sample comparison can be hampered due to collection bias. The distribution of viral genetic material might not be homogenous, and the site and intensity of sampling influence the RNA load on the specimen. To reduce intercollector bias in this study, only two veterinarians took the oropharyngeal cytobrush samples. Additionally, all people involved in the sample collections were blinded towards the vaccination status of the cats. To conclude, FCV F9 vaccination in the present study reduced the viral RNA loads shed from the oropharynx but no difference in duration and frequency of shedding replication-competent FCV was detected between the groups after experimental infection.

The second experimental FCV infection with a different field isolate (FCV 27) did not cause clinical signs in any cat, and the duration and extent of oropharyngeal FCV shedding were less intense than those with the first FCV challenge (FCV 273). The FCV viral RNA loads from the oropharynx were 10–100 times lower than during the first challenge and RNAemia was not detected. While this could be due to differences between the two challenge viruses per se, which are discussed in detail below; it is also possible that the first experimental infection elicited a strong, cross-protective immune response towards the second FCV challenge virus. A significant reduction in acute clinical signs after heterologous FCV rechallenge has been shown in previous studies [39,51]. The first FCV challenge could have induced local immunity, consisting of mucosal IgA antibodies. Secreted IgA antibodies on oropharyngeal mucosa could be why the second FCV challenge did not induce clinical signs in the cats. It has been shown that cats immunized intranasally with inactivated FCV secreted more FCV-specific IgA antibodies from the oral cavity than cats immunized subcutaneously [52]. All cats in the present study were previously experimentally infected via the oronasal route. This could have led to the formation of FCV-specific mucosal IgA antibodies and subsequent protection from clinical signs after heterologous challenge. Secreted IgA antibodies on the oral and respiratory mucosa are an important defence against pathogens affecting the respiratory tract. Mucosal IgA antibodies are able to neutralise virions before entering permissive cells and therefore prevent viral replication, as has been demonstrated for other respiratory pathogens, e.g., influenza in humans [53,54]. The data from the present study support the findings of other studies that previous FCV infection can lead to a reduction of clinical signs and, as in the present study, can prevent RNAemia.

The present study found FCV RNA in the blood from 8/10 FCV infected cats after the first FCV challenge, and FCV RNAemia lasted up to 29 days. Vaccinated cats had a significantly shorter duration of RNAemia and lower FCV RNA loads in blood compared to the control cats. No study has so far described in detail the extent and duration of FCV RNAemia after FCV infection. The presence of FCV RNA in the blood has been described [55], but it has been thought that classical FCV (not virulent–systemic FCV) is an infection of the epithelial tissues that proceeds by the infection of adjacent cells and tissues, and viremia is therefore rather uncommon [55]. The feline junctional adhesion molecule A (fJAM-A) has been identified as a receptor used by FCV for entry into susceptible cells [56]. fJAM-A was found not only on cell–cell junctions of both epithelial and endothelial tissues, but also on platelets and peripheral leukocytes [56]. The receptor presence in blood cells enables the virus to enter these cells and can cause a viraemic phase. Two cats of the vaccine group (JJI1 and JJI2) were never found to be FCV RNAemic; both cats had only very mild clinical signs with total scores of 12 and 20, respectively, and a maximum score of 3, a short duration of shedding of replication-competent FCV, and haematology values that were similar to those of the other cats in the vaccine group. Additionally, in both cats (JJI1 and JJI2), similar FCV antibody titres to the other three vaccinated cats, as determined by immunofluorescence assay (IFA [57]), were detected. However, both cats (JJI1 and JJI2) showed the highest neutralising antibody titres towards FCV F9 of all vaccinated cats on day 1 before FCV Challenge I (titres of 135 and >1215 respectively). The high neutralising antibody titres before FCV Challenge I might have been protective against RNAemia. Detailed data about IFA and neutralisation assays will be presented in Spiri et al. (in preparation [58]). It is not known if FCV RNAemia is associated with higher morbidity and/or lethality in cats. For rabbit haemorrhagic disease virus, belonging to the family *Caliciviridae* and causing severe peracute haemorrhages in various organs in rabbits, viremia is commonly seen and associated with death [59]. In human norovirus, also a member of the family *Caliciviridae* and a common pathogen in human gastrointestinal disorders, RNA is usually not found in the blood of diseased but immunocompetent people who survive the infection [60]. In the current study, no association could be found between the timepoint of the cessation of shedding of replication-competent FCV or of viral RNA from direct cytobrushes and the end of RNAemia.

Even though RNAemia was found after the first FCV challenge, only clinical signs of classical FCV disease such as oral ulcerations, fever and enlarged lymph nodes were detected in the study cats. The presence of FCV RNA in feline blood therefore does not prove virulent–systemic disease and the diagnosis of a virulent–systemic form of FCV should always be made in conjunction with the clinical presentation of the animal, the epidemiological situation, RT-qPCR diagnostics from oropharyngeal and/or blood samples, and the same virus isolate present in all diseased cats [61]. To conclude, FCV RNAemia can be detected after infection with a FCV strain causing classical clinical upper respiratory tract signs. FCV F9 vaccination had a beneficial effect in terms of reducing the duration of RNAemia and the viral RNA blood loads. The presence of FCV in blood could up open new and not yet considered transmission opportunities, e.g., through blood-sucking arthropods, which should be considered in the future [62].

We also performed complete haematology workups throughout the study period to monitor the cats. The higher lymphocyte count of the vaccinated animals shortly before and after the second injection of Vaccination I probably reflects the response of the immune system towards the vaccine. However, a physiologically transient neutrophilia and lymphocytosis induced by epinephrine release due to excitement are also commonly seen in young cats [63,64], and excitement during blood collections cannot be ruled out in the present study as all collections were performed without anaesthesia but in well clicker-trained cats. The haematology after FCV Vaccination I focused on the differences between vaccinated and control cats. No comparison of reference values for juvenile cats as described in literature [64] was undertaken. Published reference values were obtained with different methods and different cat populations (breed, living environment, not SPF) as in the present study and were therefore considered not comparable.

After the first FCV experimental infection, and to a lesser extent, also after the second experimental infection, an increase in leukocytes and banded and/or segmented neutrophils and a decrease in lymphocytes and eosinophils were found. The increase in leukocytes was mainly caused by an increase in segmented neutrophils and neutrophilia together with lymphopenia was described previously after experimental FCV infection [46]. Lymphopenia is a common sign of acute viral infections [46] and different pathological mechanisms could explain this finding. (1) Direct lympholysis by the virus is possible as the fJAM-A receptor required for viral entry can be found on peripheral leukocytes. However, it has been shown that T-lymphoblastoid cell lines can be infected by FCV in vitro and no cytopathic effect was observed [65]. Whether in vivo FCV causes a cytopathic effect in these blood cells, as is typically seen in epithelial cells, remains unknown. (2) A higher frequency of apoptosis of lymphocytes could explain the lymphopenia. This has also been detected in influenza H5N1 virus infection in humans [66]. (3) Viral infections induce inflammatory cytokines that suppress lymphopoiesis in the bone marrow [43]. (4) Changes in blood lymphocyte cell counts often reflect different distribution and not changes in production. The lymphopenia, the eosinopenia, and the neutrophilia (segmented and banded) after FCV Challenge I were only transient and median values for both groups were already within the reference range by day 3. This indicates a shift from the circulating to other pools than lympholysis, apoptosis or bone marrow suppression as the main reason for the lymphopenia shortly after experimental infection. Neutrophil counts were significantly lower in the vaccinated animals on day 6 after the first FCV infection, indicating a beneficial effect of the vaccination on haematological changes. Furthermore, the first experimental infection also seems to have a beneficial effect as the haematological changes seen after the second infection were to a lesser extent and no banded neutrophils could be detected in nonvaccinated cats.

We also determined the acute phase reaction in these cats after the FCV challenge infections. The acute phase response is a part of the innate immune system and mediates systemic inflammatory effects such as fever or leukocytosis. The acute phase protein SAA is one of the most sensitive acute phase proteins for cats [67] and increases in the early stage of various diseases at a dramatic rate [68]. Marked increases in SAA were found in cats with clinical feline infectious peritonitis [67,69]. How SAA is affected by FCV infection and FCV-induced disease is yet unknown. In 2020, Yuki et al. [67] described a significant increase in SAA concentrations in cats with upper respiratory tract infections and pneumonia, but did not state which pathogens were involved in the infections. In the present study, we describe for the first time how SAA is affected by two experimental FCV infections in vaccinated and control cats. SAA increased in all cats immediately after FCV Challenge I, with peak values on day 3 to day 6, where unspecific clinical signs such as enlarged lymph nodes and fever were observed. Oral ulcerations started to be present from day 6 to day 8, when SAA levels had already decreased in most cats. Therefore, elevated SAA values in combination with unspecific clinical signs should also raise suspicion of infectious diseases such as FCV, even if typical clinical signs are still absent. Furthermore, in the early phase of FCV infection, inflammation could be missed if only the leukogram was assessed but not SAA as hallmarks of inflammation; leukocytosis and neutrophilia were only present very mildly on day 1 after infection. Interestingly, the peak values of FCV RNAemia coincide with the peak values of SAA; both were detected on day 6 after FCV Challenge I. The second FCV challenge led to a transient increase of SAA in 9/10 cats, even though no clinical signs were present. However, the increase of SAA was to a lesser extent and earlier than after the first challenge, and no differences were observed between the group. Vaccinated cats were faster in increasing their SAA response after FCV Challenge I, but they also had significantly lower values on days 6 and 9 compared to the control cats, which indicates a beneficial effect of the vaccination. The unspecific innate defence by SAA seems to play an important role as an early immune response, even though a pre-existing adaptive immunity was already present in all vaccinated cats before FCV Challenge I and in all cats before FCV Challenge II.

Looking at the individual cats and their shedding patterns for replication-competent FCV, cat JJH3 stands out as having the longest shedding period after the first experimental infection (at least until day 63) and after the second experimental infection (at least until day 15), despite being vaccinated. The clinical signs in this cat were relatively mild and resolved within the same time, as observed in the other cats after the first experimental infection. No factors on the host side could be identified that explain this long shedding period. Before FCV Challenge I, the IFA titre and the neutralising antibody titre towards FCV 273 of cat JJH3 were similar to the other vaccinated cats. Interestingly, after FCV Challenge I, cat JJH3 showed the highest antibody titre (10,240 on day 50), determined by the IFA of all cats over the time course of the FCV infection, and the neutralising antibody titres towards FCV 273 and FCV F9 was at a similarly high level as in the other cats [58]. Before FCV Challenge II, the IFA antibody titre of cat JJH3 was similar to the other vaccinated cats and neutralising antibodies towards FCV 27 could be detected, but at a low level (titre 15). Detailed data about IFA and neutralisation assays will be presented elsewhere [58]. Overall, the findings of the antibodies determined by IFA and neutralisation do not fully explain the long FCV shedding period of cat JJH3 after both FCV challenges. More likely, a fast viral evolution that enabled the virus to evade the host immune response could be the reason for the extended shedding period. To assess that, sequencing of the virus isolates obtained over time and a corresponding neutralisation assay towards the potentially newly identified FCV variants would be needed. In general, the FCV shedding period seems to vary from cat to cat.

After FCV Challenge II, in one cat (JJI2), neither cell culture supernatants [8] nor oropharyngeal cytobrushes tested FCV-positive. This raises the question of whether the challenge was unsuccessful in this cat. The SAA values of cat JJI2 were completely unremarkable, which could support an unsuccessful second challenge infection. However, when testing this cat for the antibody response to FCV using immunofluorescence [57], a titre increase (from 2560 to 5120) was observed after FCV Challenge II (detailed data will be presented elsewhere [58]). Thus, we are confident that the challenge infection was successful in this cat. Cat JJI2 was very mildly affected by the first challenge and no RNAemia was detectable and a good local immunity with mucosal IgA could have neutralised the challenge virus before a systemic infection was possible, as described above. Interestingly, cat JJI2 showed constant, mild eosinophilia before and after FCV Challenge II. In humans, it has been shown that eosinophils can have antiviral activity [70] but if or to what extent the eosinophilia contributed to the viral defence in cat JJI2 remains inconclusive.

The difference in infection outcome concerning viral replication and clinical signs after FCV Challenge I and II was obvious. The ability of sera raised against the vaccine strain FCV F9 to neutralise FCV 27 in vitro was slightly higher than against FCV 273, but the titres against both isolates were below 1:16. It is still not known which neutralisation titre correlates with disease protection. Povey and Ingersoll [51] suggested that a titre of 1:16 or above indicates protection and a titre of 1:7 or below indicates susceptibility towards a challenge with heterologous FCV strains. To determine the in vivo infectivity of FCV 27 without the interference of pre-existing immunity, an experimental infection of FCV-naïve SPF cats would be necessary. The infectivity of both challenge isolates (inoculum) was retested and confirmed in vitro immediately after each challenge with a viral aliquot that was treated the same way as the challenge doses. On CRFK cell culture, both isolates were as infectious as determined previously (see Section 2.3 and [8]) and the infectivity was similar for both isolates. Both cats in which the challenge viruses originated showed similar clinical signs, consisting of oral ulcerations and/or gingivostomatitis. However, FCV 273 could be detected by RT-qPCR in a cytobrush sample from the oropharynx, while FCV 27 was only detectable after amplification using cell culture, indicating that the viral load for FCV 27 was also lower than that of FCV 273 in the oropharynx of the original cat. Of note for the current study, comparable inoculation doses of both viruses were given for both challenges; thus, the different outcomes should not be related to the different viral doses. Nonetheless, it seems unlikely that the different outcome of the second challenge is solely based on divergent virus characteristics, but to what extent the pre-existing immunity or the virus itself contributed to the outcome remains inconclusive. Generally, broad heterogeneity in virulence among FCV strains is observed.

## 5. Conclusions

FCV F9 vaccination of naïve cats reduced viral RNA loads, the duration of RNAemia, inflammation and the severity of clinical signs after heterologous experimental FCV infection with two FCV field strains. FCV F9 vaccination itself did not cause any clinical signs, and vaccine virus shedding was not detected after vaccinations. After FCV challenge, FCV RNA could be detected in the blood of cats showing clinical signs compatible with classical upper respiratory FCV infection, and not only in cats with virulent–systemic disease. Measurements of the acute phase response in cats with suspected recent FCV exposure can be helpful for detecting signs of the very early phase of a FCV infection prior to changes in leukogram and the occurrence of typical clinical signs. The protective and beneficial effect of the FCV F9 vaccine has been confirmed, with the present study using a current FCV F9 vaccine and a current FCV field strain.

## Figures and Tables

**Table 1:** Virus neutralisation of the two challenge viruses against the FCV vaccine strain F9.

Virus Neutralisation Against FCV F9		
Antisera	S1 ^1^	S2 ^1^
Neutralising antibody titre ^2^		
FCV 273	<5	5
FCV 27	15	15

^1^ Antisera were produced by infecting two SPF cats with FCV F9 (S1, S2). ^2^ The neutralising antibody titre reflects the reciprocal of the highest serum dilution at which at least 50% of the cell culture wells did not show a cytopathic effect.

**Table 2:** Clinical score sheet as described by Rong et al., 2014 [31].

Clinical Sign	Timepoint of Occurrence (Day Post Challenge)	Score
Sneezing occasional		1 (each day)
Sneezing persistent		2 (each day)
Dyspnoea audible rales		2 (each day)
Dyspnoea coughing		2 (each day)
Open mouth breathing		2 (each day)
Oral ulcer (single, small < 4 mm diameter)	1–5	2
	6–9	3
	≥10	4
Oral ulcers (multiple, small < 4 mm diameter)	1–4	3
	5–8	5
	≥9	7
Oral ulcers (large, ≥ 4 mm diameter)	1–4	5
	5–8	7
	≥9	9
Dehydration	1–2	3
	≥3	4
External ulcers on lips or nares, nonbleeding		4 (each day)
External ulcers on lips or nares, bleeding		6 (each day)
Conjunctivitis—serous discharge	1–3	1
	≥4	2
Conjunctivitis—mucopurulent discharge	1–2	2
	3–5	4
	≥6	6
Rhinitis—serous discharge	1–3	1
	≥4	2
Rhinitis—mucopurulent discharge	1–2	2
	3–5	4
	≥6	6
Rectal temperature	<37.2 °C	2 (each day)
	39.4–39.9 °C	1 (each day)
	40.0–40.5 °C	2 (each day)
	>40.5 °C	3 (each day)
Anorexia		1 (each day)
Death		15

**Table 3:** Time points of sample collection and assessment of clinical sings after FCV Vaccination I and II and FCV Challenges I and II (days).

	FCV Vaccination I *	FCV Challenge I	FCV Vaccination II	FCV Challenge II
Clinical examination	−1, 1, 2, 3, 4, 5, 6, 7, 13, 20, 22, 25 and 27, then weekly until day 118 after injection I	−1, 1, 2, 3, 4, 5, 6, 7, 8, 9, 10, 11, 12, 13, 14, 15, 16, then twice a week until day 108 after Challenge I, then weekly until FCV Vaccination II	−1, 1, 2, 3, 4, 5, 6, 7, 8 and 9, then weekly until FCV Challenge II	−1, 1, 2, 3, 4, 5, 6, 7, 8, 9, 10, 11, 12, 13, 14, 15 and 16, then twice a week until day 64, then weekly until day 120 after FCV Challenge II
Clinical scoring	-	1, 2, 3, 4, 5, 6, 7, 8, 9, 10, 11, 12, 13, 14 and 15	-	1, 2, 3, 4, 5, 6, 7, 8, 9, 10, 11, 12, 13, 14 and 15
Oropharyngeal cytobrushes	−1, 1, 2, 3, 4, 5, 6, 7, 13, 20, 22, 25 and 27, then weekly until day 118 after injection I	−4, 1, 2, 3, 4, 5, 6, 7, 8, 9, 10, 11, 12, 13, 14, 15, 16 and 22, then weekly until day 106 after FCV Challenge I	−1, 1, 2, 3, 4, 5, 6, 7, 8 and 9, then weekly until FCV Challenge II	−4, 1, 2, 3, 4, 5, 6, 7, 8, 9, 10, 11, 12, 13, 14, 15 and 16, then weekly until day 42 after FCV Challenge II
EDTA blood collection	−1, 4, 7, 13, 20, 22 and 27, then weekly until day 96 and on day 200 after injection I	−1, 1, 2, 3, 6, 9, 13, 16, 22, 29, 36 and 43, then weekly until day 106 and day 134, 162 after FCV Challenge I	−1, 2, 3 and 8	−1, 1, 3, 6, 9, 13 and 16, then weekly until day 44 after FCV Challenge II
Serum collection	-	−1, 1, 3, 6, 9, 13 and 16	-	−1, 1, 3, 6 and 9

* FCV Vaccination I consisted of two injections (injections I and II) 21 days apart.

## Data Availability

All available data are presented in this manuscript.

## Appendix A

The results of the Friedman and Dunn’s post-tests of the white blood cell differentiation, the body temperature, and the serum amyloid-A (SAA) after FCV Challenge I are shown in Table A1.

**Table A1:** Summary of the white blood cell differentiation, body temperature and SAA variation over time after FCV Challenge I.

	**Change Over Time (Friedman and Dunn’s Post Tests)** **Vaccinated Group**	**Change Over Time (Friedman and Dunn’s Post Tests)** **Control Group**
Leukocytes	PF < 0.0001 Day 1 ↑/Day 6 (PD = 0.0011) Day 1 ↑/Dax 9 (PD = 0.0014) Day 1 ↑/Day 13 (PD = 0.008) Day 1 ↑/Day 162 (PD = 0.0038) Day 6 ↓/Day 78 (PD = 0.0122) Day 9 ↓/Day 78 (PD = 0.0151) Day 78 ↑/Day 162 (PD = 0.0371)	PF = 0.0012 Day 1 ↑/Day 6 (PD = 0.0042) Day 1 ↑/Day 162 (PD = 0.003)
Total (segmented and banded) neutrophils	PF = 0.0003 Day 1 ↑/Day 9 (PD = 0.001) Day 1 ↑/Day 13 (PD = 0.025) Day 3 ↑/Day 9 (PD = 0.0072)	PF = 0.0005 Day 1 ↑/Day 99 (PD = 0.0042) Day 1 ↑/Day 106 (PD = 0.0305) Day 1 ↑/Day 162 (PD = 0.008)
Banded neutrophils	PF = 0.0015 Significance lost in the post tests	PF = 0.0012 Day −1/Day 1 ↑ (PD = 0.016) Day 1 ↑/Day 16 (PD = 0.016)
Lymphocytes	PF < 0.0001 Day −1 ↑/Day 1 (PD = 0.0034) Day −1 ↑/Day 3 (PD = 0.0042) Day −1 ↑/Day 6 (PD = 0.0336) Day 1 ↓/Day 50 (PD = 0.011) Day 1 ↓/Day 78 (PD = 0.0065) Day 3 ↓/Day 50 (PD = 0.0136) Day 3 ↓/Day 78 (PD = 0.008)	PF < 0.0001 Day 1 ↓/Day 22 (PD = 0.0122) Day 1 ↓/Day 50 (PD = 0.0409) Day 1 ↓/Day 56 (PD = 0.0336) Day 3 ↓/Day 22 (PD = 0.0496) Day 6 ↓/Day 22 (PD = 0.0226)
Eosinophils	PF < 0.0001 Day 1 ↓/Day 78 (PD = 0.0034) Day 3 ↓/Day 78 (PD = 0.0058) Day 6 ↓/Day 78 (PD = 0.0014) Day 9 ↓/Day 78 (PD = 0.0052) Day 13 ↓/Day 78 (PD = 0.0305)	PF < 0.0001 Day 6 ↓/Day 43 (PD = 0.0047) Day 6 ↓/Day 63 (PD = 0.0305) Day 6 ↓/Day 78 (PD = 0.0336) Day 9 ↓/Day 43 (PD = 0.0450)
Body temperature	PF < 0.0001 Day 3 ↑/Day 13 (PD = 0.021) Day 4 ↑/Day 13 (PD = 0.0122) Day 5 ↑/Day 13 (PD = 0.0184) Day 3 ↑/Day 14 (PD = 0.0354) Day 4 ↑/Day 14 (PD = 0.021) Day 5 ↑/Day 14 (PD = 0.0311)	PF = 0.0004 Day 4 ↑/Day 13 (PD = 0.021) Day 4 ↑/Day 14 (PD = 0.021) Day 5 ↑/Day 13 (PD = 0.0354) Day 5 ↑/Day 14 (PD = 0.0354)
SAA	PF = 0.002 Day 3 ↑/Day 13 (PD = 0.016) Day 3 ↑/Day 16 (PD = 0.0269) Day 6 ↑/Day 13 (PD = 0.0269) Day 6 ↑/Day 16 (PD = 0.044)	PF < 0.001 Day 6 ↑/Day −1 (PD = 0.007) Day 6 ↑/Day 13 (PD = 0.0093) Day 6 ↑/Day 16 (PD = 0.0022)

The results of the Friedman and Dunn’s post-tests of the white blood cell differentiation after FCV Challenge II are shown in Table A2.

**Table A2:** Summary of white blood cell differentiation variation over time after FCV Challenge II.

	**Change Over Time (Friedman and Dunn’s Post Tests)** **Vaccinated Group**	**Change Over Time (Friedman and Dunn’s Post Tests)** **Control Group**
Leukocytes	P_F_ = 0.0032 Day 3 ↓/Day 44 (P_D_ = 0.0392) Day 16 ↓/Day 44 (P_D_ = 0.0276)	P_F_ = 0.0002 Day 1 ↑/Day 3 (P_D_ = 0.0051) Day 1 ↑/Day 6 (P_D_ = 0.0276) Day 1 ↑/Day 13 (P_D_ = 0.016)
Total (segmented) neutrophils	Not significant	P_F_ = 0.0098 Day 1 ↑/Day −1 (P_D_ = 0.023) Day 1 ↑/Day 6 (P_D_ = 0.0392) Day 1 ↑/Day 13 (P_D_ = 0.0392)
Lymphocytes	P_F_ < 0.0001 Day 1 ↓/Day 29 (P_D_ = 0.0062) Day 1 ↓/Day 36 (P_D_ = 0.0392) Day 3 ↓/Day 29 (P_D_ = 0.0091)	P_F_ < 0.0001 Day 1 ↓/Day 36 (P_D_ = 0.016) Day 1 ↓/Day 44 (P_D_ = 0.0075) Day 3 ↓/Day 29 (P_D_ = 0.023) Day 3 ↓/Day 36 (P_D_ = 0.0051) Day 3 ↓/Day 44 (P_D_ = 0.0023)
Eosinophils	P_F_ = 0.0034 Significance lost in the post test	Not significant
SAA	P_F_ = 0.0118 Day −1 ↓/Day 3 (P_D_ = 0.027)	P_F_ = 0.0117 Day −1 ↓/Day 3 (P_D_ = 0.0194) Day 3 ↑/Day 9 (P_D_ = 0.0373)
